# Complete Genome Sequence of the Carbazole Degrader *Pseudomonas resinovorans* Strain CA10 (NBRC 106553)

**DOI:** 10.1128/genomeA.00488-13

**Published:** 2013-07-25

**Authors:** Masaki Shintani, Akira Hosoyama, Shoko Ohji, Keiko Tsuchikane, Hiromi Takarada, Atsushi Yamazoe, Nobuyuki Fujita, Hideaki Nojiri

**Affiliations:** Biotechnology Research Center, The University of Tokyo, Bunkyo-ku, Tokyo, Japana; Department of Applied Chemistry and Biochemical Engineering, Graduate School of Engineering, Shizuoka University, Naka-ku, Hamamatsu, Shizuoka, Japanb; Biological Resource Center, National Institute of Technology and Evaluation, Nishihara, Shibuya-ku, Tokyo, Japanc; Agricultural Bioinformatics Research Unit, Graduate School of Agricultural and Life Sciences, The University of Tokyo, Bunkyo-ku, Tokyo, Japand

## Abstract

*Pseudomonas resinovorans* strain CA10 can grow on carbazole as its sole carbon and nitrogen source. Here, we report the complete nucleotide sequence of the CA10 genome (a 6,285,863-bp chromosome and a 198,965-bp plasmid). CA10 carries a larger number of genes that are potentially responsible for aromatic hydrocarbon metabolism than do other previously sequenced *Pseudomonas* spp.

## GENOME ANNOUNCEMENT

*Pseudomonas resinovorans* strain CA10 (NBRC 106553) was isolated from activated sludge and can utilize carbazole, a nitrogen heteroaromatic compound, as its sole carbon, nitrogen, and energy source (1, 2). Strain CA10 possesses about 200 kb of the incompatibility group (Inc) P-7 plasmid pCAR1 (3, 4), whose full sequence was determined previously (5, 6). Carbazole is converted to catechol via anthranilate by plasmid-encoded Car and Ant enzymes (1), and the catechol is further degraded by the chromosomally encoded enzymes (1, 3, 7).

The genome of CA10 was determined using a combined strategy of GS FLX Titanium and HiSeq 1000 technologies. Two different types of libraries were constructed for sequencing: a standard library (600 to 900 bp) for GS FLX Titanium and a paired-end library (200 to 500 bp) for HiSeq 1000. A total of 117,465,953 base sequences (276,857 reads) were obtained with a depth of 18-fold genome coverage by the GS FLX Titanium system, and 487,867,241 base paired-end sequences (5,396,359 reads) were obtained with a depth of 75-fold genome coverage by the HiSeq 1000 system. The assembly was performed using Phred (8, 9) and Newbler v2.6 software (10). Ambiguities in the sequences were manually inspected and corrected using Illumina sequence reads. Closing of gaps was accomplished by (i) local assembly with the aid of Sanger reads of end sequences from fosmid libraries with inserts of 30 kb in pCC1FOS (Epicentre Biotechnologies, Madison, WI), (ii) filling with additional reads by primer walking, (iii) sequencing of fosmid clones with random insertions generated by the Template Generation System II kit (Finnzymes, Vantaa, Finland), and (iv) sequencing of PCR products using an ABI 3730 sequencer.

The genome of CA10 consists of a circular chromosome (6,285,863 bp; 65.6% G+C content) and a circular plasmid named pCAR1.3 (198,965 bp; 56.4% G+C content). pCAR1.3 is shorter than the previously reported pCAR1 and pCAR1.2 plasmids (GenBank/EMBL/DDBJ accession no. AB088420 and AB474758, respectively), because one of the 1,261-bp DNA regions containing *carAa*, which was duplicated on pCAR1 and pCAR1.2 (i.e., these plasmids carry two copies of *carAa*), was deleted on pCAR1.3. Complete sequences of the CA10 genome were analyzed using MIGAP (http://www.migap.org/) for predicting protein-coding, tRNA, and rRNA genes. Their functional annotations were assigned using UniProt, InterPro, Hamap, and an in-house database composed of manually curated microbial genome sequences (S. Miyazawa, S. Isaki, N. Ichikawa, and N. Fujita, unpublished data). The CA10 chromosome has 5,652 coding sequences (CDSs), five rRNA operons, and 70 tRNA genes. The anthranilate-degradating genes *antABC* and *antR* were found not only on pCAR1.3 but also on the chromosome, as was predicted previously (11). Comparisons between CA10 and the other 14 previously sequenced *Pseudomonas* strains using the RAST server (http://rast.nmpdr.org/) (12) showed that CA10 carries a larger number of CDSs for metabolism of aromatic compounds (241 CDSs) than do the other strains (49 to 187 CDSs). This suggests that the CA10 may be suitable for the metabolism of various aromatic compounds as the original host of pCAR1.

### Nucleotide sequence accession numbers.

The nucleotide sequences of the CA10 chromosome and pCAR1.3 were deposited in the DDBJ/EMBL/GenBank databases under the accession no. AP013068 and AP013069, respectively.
